# Draft Genome Sequence of *Micromonospora* sp. Strain WMMB235, a Marine Ascidian-Associated Bacterium

**DOI:** 10.1128/genomeA.01369-16

**Published:** 2017-01-12

**Authors:** Navid Adnani, Doug R. Braun, Bradon R. McDonald, Marc G. Chevrette, Cameron R. Currie, Tim S. Bugni

**Affiliations:** a Pharmaceutical Sciences Division, University of Wisconsin-Madison, Madison, Wisconsin, USA; b Department of Bacteriology, University of Wisconsin-Madison, Madison, Wisconsin, USA; c Laboratory of Genetics, University of Wisconsin-Madison, Madison, Wisconsin, USA

## Abstract

*Micromonospora* sp. strain WMMB235 was isolated in 2011 off the coast of the Florida Keys, USA, from a marine ascidian as part of an ongoing drug discovery project. Analysis of the ~7.1-Mb genome provides insight into this strain's biosynthetic potential, means of regulation, and response to coculturing conditions.

## GENOME ANNOUNCEMENT

*Micromonospora* spp. have long been recognized as crucial sources of antibiotics (1). The aminoglycoside antibiotics gentamicin (2) and netilmicin (3), antitumor antibiotics lomaiviticins A and B (4), tetrocarcins (5–8), LL-E33288 (9), anthracycline antibiotics (10), the anthraquinone lupinacidins A to C (11, 12) and diazepinomicin, an antimicrobial marine alkaloid (13), are but a few of the medicinally significant secondary metabolites produced by *Micromonospora* spp.; members of the genus have been credited with providing over 700 compounds of medicinal value (1). Despite this, relative to other actinomycetes, there is a scarcity of genome information on *Micromonospora*.

*Micromonospora* spp. are Gram-positive, generally aerobic, and tend to exhibit complex life cycles, differentiating into both substrate mycelia and spores, although aerial mycelia are not a common feature (14). The life cycle characteristics, habitats, and both past and putative future applications of these bacteria have been excellently reviewed; notable emphasis now focuses on their use in biofuel production (15).

To identify new and otherwise cryptic biosynthetic gene clusters and their corresponding bioactive natural products through coculturing methodologies, we recently carried out metabolomics studies involving *Micromonospora* sp. strain WMMB235 in the presence of *Rhodococcus* sp. WMMA-185. *Micromonospora* sp. WMMB235 was isolated in 2011 from a marine-associated ascidian collected off the coast of the Florida Keys.

The complete genome of *Micromonospora* sp. WMMB235 was sequenced at the Duke Center for Genomic and Computational Biology (GCB) using PacBio RSII (Pacific Biosciences) technology. Reads were constructed using the HGAP assembler (16) into two different contigs that were 7.02 Mb and 14.7 kb in size, respectively. We hypothesize that the smaller of the two contigs is a plasmid, whereas the larger contig represents the full circular chromosome of WMMB235. This logic is supported by a ≈10-kb overlap of the ends of the contig. Within this overlap are five single-base gaps that we have not been able thus far to resolve. The smaller 14-kb contig aligns well with the 3′ end of the larger contig, with the notable exception that it contains a 1,402-bp insert from elsewhere in the genome. Consequently, we do not yet know if this smaller contig represents a real variant sequence of the chromosome or is merely an assembly error.

Open reading frames were predicted by Prodigal (17) and annotated using HMMer models for the TIGRfam (18), KEGG (19, 20), and Pfam (21, 22) databases. The genome is 72.83% GC and has 90.27% coding density. The organism’s secondary metabolic content/potential was assessed on the basis of antiSMASH (23, 24), PRISM (25), and custom pipelines. Housed within the *Micromonospora* sp. WMMB235 genome were found a single type I polyketide (PKS), a single type III PKS, one lanthipeptide system (26), and seven hybrid biosynthetic gene clusters. Thus, genome analysis of WMMB235 has revealed this *Micromonospora* to have a wealth of biosynthetic machineries at its disposal.

### Accession number(s).

This whole-genome shotgun project has been deposited at DDBJ/ENA/GenBank under the accession no. MDRX00000000. The version described in this paper is version MDRX01000000.
